# Influence of Extrusion Rate on Microstructure and Mechanical Properties of Magnesium Alloy AM60 and an AM60-Based Metal Matrix Nanocomposite

**DOI:** 10.3390/nano12152682

**Published:** 2022-08-04

**Authors:** Danai Giannopoulou, Jan Bohlen, Noomane Ben Khalifa, Hajo Dieringa

**Affiliations:** 1 Institute of Material and Process Design, Helmholtz-Zentrum Hereon, Max-Planck-Str. 1, 21502 Geesthacht, Germany; 2 Institute of Product and Process Innovation, Leuphana University Lüneburg, Universitätsallee 1, 21335 Lüneburg, Germany

**Keywords:** hot extrusion, magnesium, AM60, MMNC, AlN nanoparticles, texture

## Abstract

Metal matrix nanocomposites are attracting attention because of their great potential for improved mechanical properties and possible functionalization. These hybrid materials are often produced by casting processes, but they can also develop their property profile after hot working, e.g., by forging or extrusion. In this study, a commercial cast magnesium alloy AM60 was enriched with 1 wt.% AlN nanoparticles and extruded into round bars with varied extrusion rates. The same process was carried out with unreinforced AM60 in order to determine the influences of the AlN nanoparticles in direct comparison. The influence of extrusion speed on the recrystallization behavior as well the effect of nanoparticles on the microstructure evolution and the particle-related strengthening are discussed and assessed with respect to the resulting mechanical performance.

## 1. Introduction

In recent years, metal matrix nanocomposites (MMNCs) have been investigated with increasing intensity as they can open up new areas of application in lightweight construction due to their often significantly improved mechanical properties compared to their matrix alloys and a very low nanoparticle content [1]. Disadvantages such as propensity for corrosion [2], limited high temperature strength and creep resistance have been overcome by reinforcing Mg alloys with particles or fibers such as ceramic nanoparticles, which offer relatively high strength to Mg alloys [1]. Although the addition of microscale particles can achieve an increase in strength, at the same time a reduction in the ductility of the composite is observed when the reinforcement concentrations are high, something that does not occur when the particles are nanoscale, which is ideal for Orowan strengthening. Moreover, the ceramic nanoparticles are stable at higher temperatures unlike precipitation-strengthened alloys, which become overaged and lose strength [1,2,3,4,5,6].

The major challenge in the production of MMNCs is the homogeneous nanoparticle distribution in metallic melts because they have high surface area and poor wettability with the melt. The key to this challenge is the use of additional forces during casting [7]. Powder metallurgical process [8], disintegrated melt deposition (DMD) [8,9] and evaporation of Mg [10] are processes that can achieve a uniform distribution of the nanoparticles in the melt, but they have disadvantages which limit their usage, such as lack of mass production, and therefore they are not commercially viable [11]. Conventional casting processes are more relevant for commercial applications but require additional forces such as electromagnetic [12,13], ultrasonic [6,14] and mechanical shearing [15,16,17] for distributing nanoparticles.

In addition to the need to choose the appropriate fabrication process, the choice of nanoparticles is equally important. AlN nanoparticles and Mg have similarities in crystal structure and lattice parameters [18]. Thus, the growth of Mg on an AlN particle is relatively easy and does not require the accommodation of large strain, and the small differences in lattice parameter can be overcome locally in the first layers of Mg that grow on the AlN particles, described in [11], which revealed the impact of AlN nanoparticle addition to an AM60 alloy. The AlN nanoparticles improved the yield strength by 103%, ultimate tensile strength by 115% and ductility by 140%. Therefore, the question arises whether ceramic nanoparticles influence recrystallization and microstructure development and if further homogenization of the nanoparticle distribution can be achieved in a subsequent deformation process such as extrusion and what effect this may have on mechanical properties of extruded profiles. Extrusion is a process which significantly changes the microstructure of the material through deformation and dynamic recrystallization.

AlN nanoparticles were distributed during casting into an AM60 Mg alloy using high-shear dispersion technology (HSDT). After casting, extrusion experiments followed with four different ram speeds. The aim of this work was to investigate how the nanoparticles influence the microstructure evolution and if there are particle-related strengthening mechanisms present. Furthermore, the effect of different extrusion speeds and nanoparticle content on recrystallization behavior was analyzed.

## 2. Materials and Methods

### 2.1. Casting

Commercial Mg alloy AM60 (6 wt.% Al, 0.2–0.4 wt.% Mn, rem. Mg) was heated to 720 °C, 1 wt.% AlN powder was added and the mixture was treated for 10 min at 1000 rpm with HSDT, performed with a rotor-stator shearing device (ZYOMAX Ltd., Middlesex, UK). The AlN nanoparticles have an average diameter of 80 nm [11], and their synthesis and properties are described in [19]. Then, a cylindrical steel mold preheated to 400 °C was filled with melt and put into a three-zone resistance furnace. The mold with the melt was lowered into a water bath underneath the furnace at a constant speed of 3 mm/s. Therefore, solidification was initiated at the bottom of the mold, and directional solidification was carried out. For comparison, the same alloy was prepared without adding AlN but also with 10 min of intensive shearing to keep the processing identical.

### 2.2. Extrusion

Four billets each of AM60 and AM60 with AlN nanoparticles were machined from the castings with a diameter of 49 mm to fit a 50 mm diameter container of the extrusion press (2.5 MN automatic extrusion setup, Müller Engineering, Todtenweis, Bavaria, Germany). Indirect extrusion trials were performed at 300 °C using different ram speeds of 0.6, 4.4, 6.6 and 8.0 mm/s to produce round bars with a diameter of 10 mm, which corresponds to an extrusion ratio of 1:25. After exiting the die, the profiles cooled down in air to room temperature.

### 2.3. Microstructure and Mechanical Characterization

A microstructural analysis was carried out by using light optical microscopy (LOM) on longitudinal sections of the profiles. Samples were cut from the extruded bars, and each sample was embedded in resin, ground (SiC papers: 500, 800, 1200, 2500), polished (0.25 µm diamond paste in OPS water free suspension) and etched using acetic picric acid solution (10 mL acetic acid, 4.2 g picric acid, 70 mL ethanol, 10 mL distilled water). The average grain size was calculated using a computer-aided linear intercept measurement. A Panalytical^TM^ X-ray goniometer setup using Cu-Kα radiation in reflection geometry was used to measure six pole figures to a tilt angle of 70° on cross sections of the extruded bars. The data were used to recalculate and present inverse pole figures parallel to the extrusion direction by applying an open access code MTEX [20]. 

HV5 Vickers hardness tests were performed on the longitudinal sections of the extruded samples with an EMCO M1C 010 test machine using an indentation load of 5 kg. The hardness results were the average of 10 indentations for each sample. Samples were machined to a gauge length of 30 mm and a diameter of 5 mm with their longitudinal axis parallel to the extrusion direction for tensile testing, while for compression tests they had a length of 13.5 mm and a diameter of 9 mm. Both tests were conducted at room temperature with a constant rate of 10^−3^ s^−1^ using a universal testing machine (Zwick^TM^ Z050).

## 3. Results

### 3.1. Extrusion

**Figure 1:**
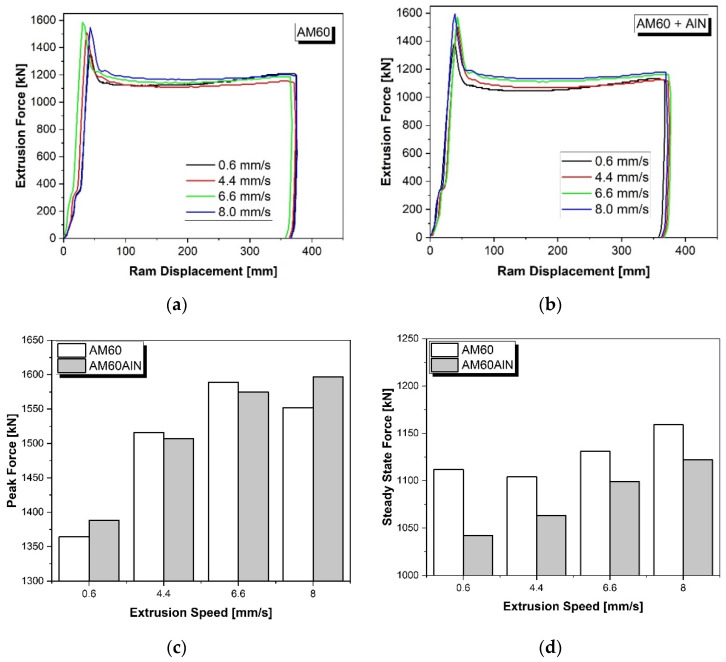
Indirect extrusion diagrams of the AM60 alloy (**b**) with and (**a**) without AlN nanoparticles. Peak forces (**c**) and steady state forces (**d**) of both materials at different extrusion speeds.

The diagrams of the indirect extrusion experiments are presented in Figure 1a,b for the AM60 alloy and the AM60 alloy with AlN nanoparticles, respectively. In the indirect extrusion process, the required forces relate to the plastic flow of the material at the onset as well as during a steady state phase. Therefore, the applied force increases to an initial peak force and then decreases continuously as the material starts to flow. The main factor affecting the material flow is the variation of the extrusion rate and the presence of AlN nanoparticles. It can be noted that the peak force increases with increasing extrusion speed; see Figure 1c. Only the AM60 extruded at 8 mm/s shows a slight decrease in peak force compared to the AM60 extruded at 6.6 mm/s. 

The differences between AM60 with and without AlN nanoparticles are only minor, and there is no tendency that would prove that the AlN nanoparticles have an influence on the peak force. The influence of the AlN nanoparticles on the steady state force, however, is significant; see Figure 1d. With nanoparticles, it is lower than without nanoparticles, and the difference is higher at low extrusion speeds than at high extrusion speeds. At the same time, the steady state force increases with increasing extrusion speed; the only exception to this tendency is for the lowest extrusion speed of 0.6 mm/s of AM60.

### 3.2. Microstructure

**Figure 2:**
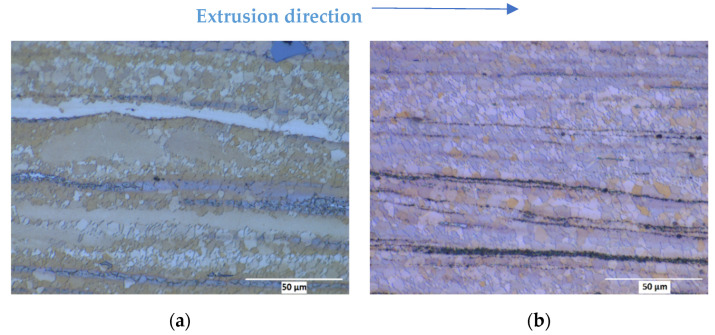

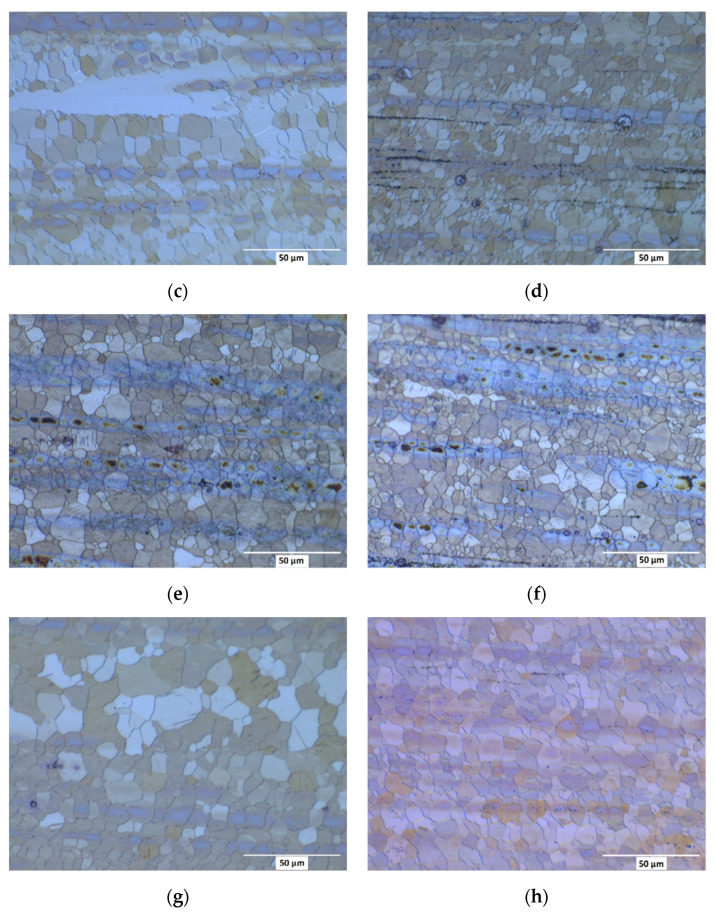
Optical micrographs from longitudinal sections of extruded round bars of AM60 (**a**,**c**,**e**,**g**) and AM60 with 1 wt.% AlN (**b**,**d**,**f**,**h**) with different extrusion speeds: 0.6 (**a**,**b**), 4.4 (**c**,**d**), 6.6 (**e**,**f**) and 8.0 mm/s (**g**,**h**).

**Figure 3:**
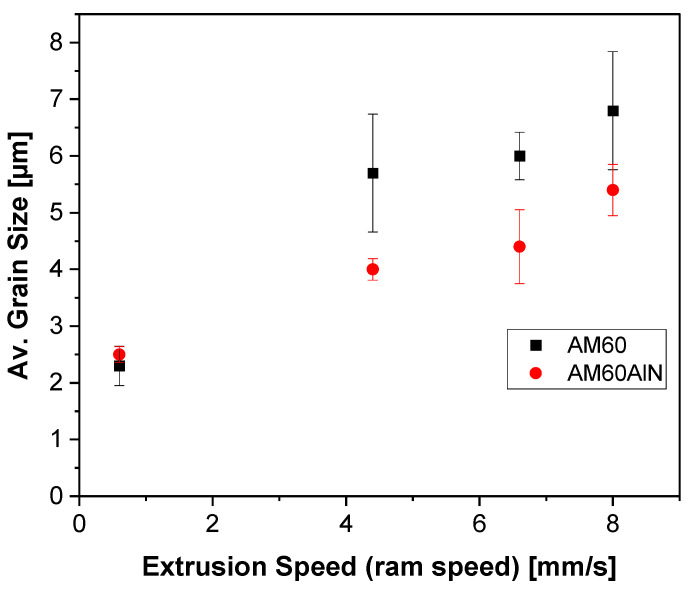
Average grain size values of extruded AM60 and AM60 + 1 wt.% AlN series.

Figure 2 and Figure 3 show the micrographs of the investigated materials after extrusion with different extrusion rates and the grain size as a function of the extrusion speed, respectively. Figure 2a,c,e,g represent the extruded samples of the AM60 alloy, while in Figure 2b,d,g,h the nanocomposites with 1 wt.% AlN are shown. Some of the AlN-containing nanocomposites show particle stringers that form horizontally through the microstructure in the direction of extrusion. AM60 extruded at 0.6 mm/s (Figure 2a) exhibits a partly recrystallized microstructure. The recrystallized area mainly consists of equiaxed fine grains, while the non-recrystallized region is composed of the remaining elongated grains parallel to the extrusion direction. With the increase in extrusion speed (Figure 2c,e,g), the degree of recrystallization increases, and the microstructure becomes homogeneous with gradually coarser grains.

In the case of the AM60 with AlN nanoparticles (Figure 2b,d,f,h) a fully recrystallized microstructure is revealed even after low speed extrusion at 0.6 mm/s. Similar to the AM60 samples, the extrusion speed influences the counterparts with added AlN nanoparticles, which reveals coarser grains with the increase in the ram speed. It is evident from Figure 3 that the AlN nanoparticle addition led to a reduction in the average grain size at higher speeds, whereas the result is comparable at the lowest speed despite the different degree of recrystallization.

### 3.3. Texture

**Figure 4:**
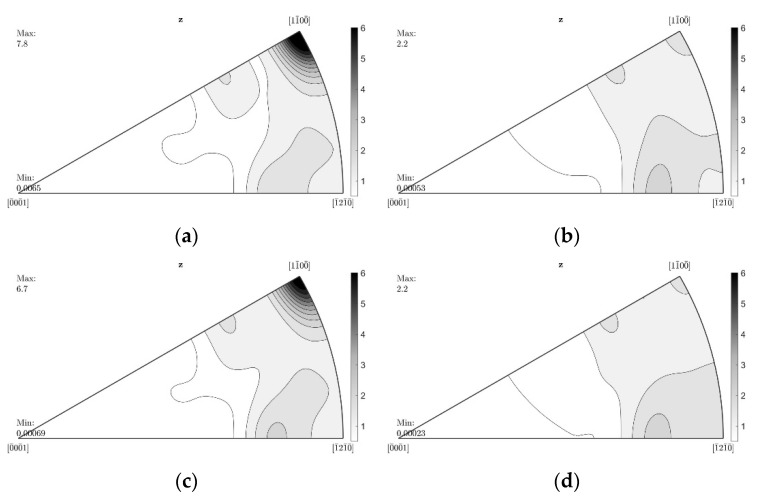

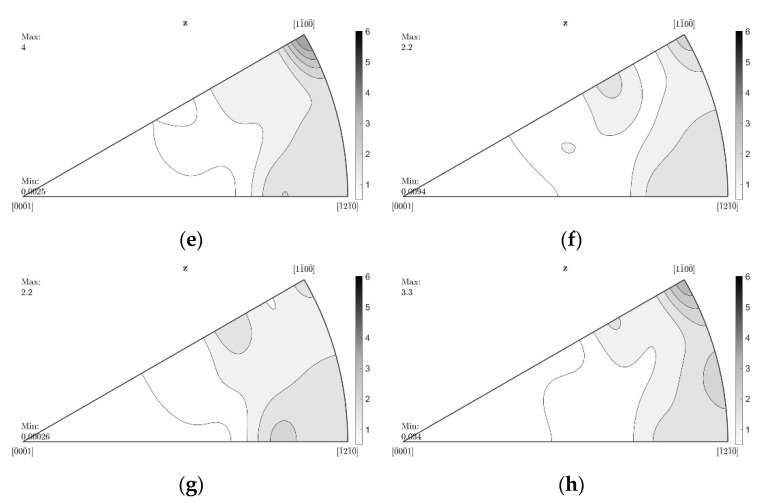
Inverse pole figures in extrusion direction from extruded round bars of AM60 (**a**,**c**,**e**,**g**) and AM60 with 1 wt.% AlN (**b**,**d**,**f**,**h**) with different extrusion speeds, left: <00.1>, bottom right: <10.0>, top right: <11.0>.

Figure 4 illustrates the textures of the extruded round bars in the form of inverse pole figures in the extrusion direction. In the case of the AM60 nanoparticle-free alloy, the texture reveals a strongly pronounced component around the <10.0> pole. This prismatic component decreases in intensity as the extrusion ram speed increases until it reaches the maximum speed at 8.0 mm/s. On the other hand, in the AM60 samples with AlN reinforcement, this component is not visible even at the low extrusion speed. A weak texture with orientations consistent with a tilt of basal planes out of the extrusion direction remains.

### 3.4. Mechanical Properties

**Figure 5:**
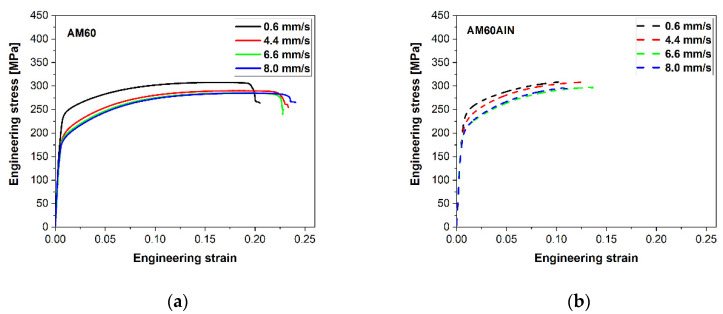

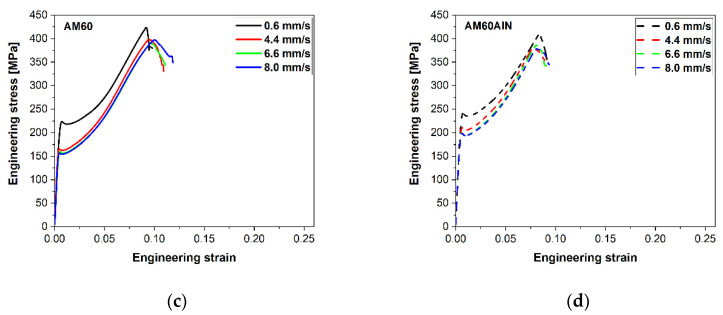
Stress–strain diagrams of extruded bars of AM60 (**a**,**c**) and AM60 with 1 wt.% AlN (**b**,**d**) in tension (**a**,**b**) and compression (**c**,**d**).

Stress–strain diagrams from tension and compression tests parallel to extrusion direction at room temperature are presented in Figure 5, and the corresponding mechanical properties are summarized in Table 1. Each test was repeated five times, and the mechanical properties were calculated by averaging the test results.

In the case of the pure AM60-based series, a large variation between the slowly extruded AM60 alloy (0.6 mm/s) and the faster extruded samples (4.4, 6.6, 8.0 mm/s) can be seen. AM60 extruded at 0.6 mm/s exhibits the highest TYS, UTS, CYS and UCS values while revealing the lowest values in fracture strain. As the extrusion ram speed increases (4.4, 6.6, 8.0 mm/s), the stress levels in tension and compression decrease continuously; however, the fracture strain remains comparable between 22 and 24% for these extrusion conditions. 

For the AM60 nanocomposites, again, the slowly extruded sample reveals highest TYS, UTS, CYS and UCS values, but a more ductile behavior is no longer observed. It is noteworthy that the addition of the AlN nanoparticles strengthens the AM60 alloy in almost all extrusion conditions. In the case of the AM60AlN extruded at 0.6 mm/s, it is obvious, that the tension and compression curves remains at similar stress levels to the corresponding AM60 sample extruded at 0.6 mm/s without AlN nanoparticles; see Table 1. The variation between the AM60AlN extruded at 0.6 mm/s and the remaining nanocomposites is not as pronounced as in the case of the pure AM60-based series. In the samples extruded at higher ram speed (4.4, 6.6 and 8.0 mm/s), the stress values decrease as the ram speed increases. Specifically, when the extrusion ram speed reaches the value of 8.0 mm/s, no further reduction is observed in TYS and CYS, and UTS and UTS remain roughly the same. Simultaneously, a distinct impact on the fracture strain develops in tension with the addition of the nanoparticles as the AM60AlN-based series stops exhibiting ductile behavior.

**Figure 6:**
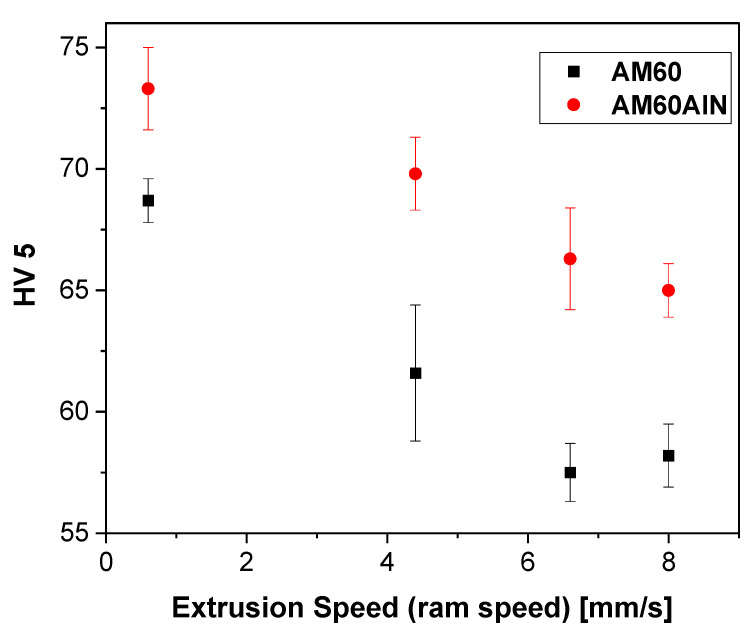
Hardness of extruded AM60 and AM60 + 1 wt.% AlN series.

Figure 6 shows the hardness measurements of the extruded AM60 and AM60AlN series, parallel to the extrusion direction, as a function of the extrusion ram speed. It appears that the AlN nanoparticles reinforce the AM60 samples with a noticeable incensement in hardness levels. Moreover, the tendency of the AM60AlN series is similar to the CYS results in Table 1, and the hardness levels decrease as the ram speed increases.

## 4. Discussion

### 4.1. Extrusion

The initiation of the plastic deformation during extrusion is expressed as the peak force that increases as the extrusion speed increases; see Figure 1c. This applies to the AM60 and the AM60 with AlN nanoparticles. While the peak force is not systematically varied with the addition of AlN particles to AM60, there is no fundamental change revealed at this initiating point of plastic flow. In contrast, there is a comprehensive decrease visible in the steady state flow stress for the particle-modified alloy. Thus, the AlN nanoparticle addition does not lead to strengthening of the material during steady state flow but rather to a reduction in the flow stress. As this effect is unlikely to be driven by the deformation behavior which is carried by dislocation glide, it can be hypothesized that an impact of the recrystallization behavior plays a role, which is addressed during the discussion of the microstructure and texture development in the following.

### 4.2. Microstructure and Texture

The increasing extrusion ram speed in both the AM60 alloy and AM60AlN nanocomposite leads directly to a fully recrystallized microstructure; see Figure 2. However, this effect is mostly visible in the AM60 alloy with a transition from a partly recrystallized microstructure to a fully recrystallized microstructure with increasing extrusion speed. This tendency is associated with an increase in the forming heat and a concurrent increase in the temperature in the extrusion die enabling a diffusion-related enhancement of the microstructure development [21]. In the case of the AM60AlN nanocomposite, the microstructure appears fully recrystallized at the lowest speed and therefore appears further developed in this regard compared to its AlN-free counterpart. In contrast, the microstructure of the AM60 extruded samples develops into a slightly coarser-grained microstructure at the higher extrusion speeds. It is evident that the result is comparable at the lowest speed despite the different degrees of recrystallization. This extensive recrystallized behavior is consistent with an increase in the grain nucleation rate due to the AlN addition as well as a concurrent retardation in the grain growth, hypothetically due to the higher grain nucleation rate. Note that this development is consistent with the decrease in the force levels during extrusion as mentioned above. 

In Mg extrusion, partially recrystallized microstructures exhibiting strong textures, dominated by a <10.0> fiber component, are common [22]. The inverse pole figures of the extruded AM60 alloy round bars introduce such a strongly pronounced component around the prismatic pole in the samples extruded with lower speeds. On the other hand, the texture of the AM60 alloy with AlN reinforcements appears stable with no distinct changes. At lower extrusion rates, the texture does not have the dominant prismatic component that is found in the AM60 alloy textures. According to the development of the microstructure of nanocomposites, the texture does not appear to have the dominant prismatic component at lower speeds. Hence, the texture evolution and simultaneously the state of complete recrystallization are shifted to low extrusion rates due to the addition of AlN. Although the extrusion processes in both the AM60 alloy and AM60AlN nanocomposite do not demonstrate dramatic differences, the microstructure development is accelerated in the sense of an earlier evolution of a fully recrystallized microstructure at low forming rates. Note that for the AlN-containing AM60, the strong prismatic component is more significant again at highest extrusion speeds, which does not correspond to a distinct grain size development in the microstructure. This is obviously not associated with a more pronounced grain growth and a concurrent preferential development of stable oriented grain orientations but rather relates to the original grain nucleation orientation that seems to stabilize at these original orientations if the nucleation rate increases [23]. In any case, the stabilization of the microstructure evolution during the massive forming process of indirect extrusion is evident.

### 4.3. Mechanical Properties

The significant reduction in ductility of the nanoparticle-reinforced AM60 compared to the AM60 without particles in tensile testing may be due to the presence of particle agglomerates that are still present in the matrix. These agglomerates, which can also be seen as particle stringers in Figure 2b,d,f,h, may have a crack-initiating effect in the tensile test during plastic deformation, as no transmission of the stress is provided at these points. 

Various models describe the increase in yield strength in nanocomposites. Grain-size-related (Hall–Petch relationship, Equation (1)) and particle strengthening (Orowan, Equation (2)) contributions were considered in the present work in order to understand the strengthening mechanisms. In Equation (1), *σ_y_* is the yield stress; *σ_0_* is the friction stress that, without any strengthening mechanisms, allows dislocations to move on slip planes in a single crystal; *k_y_* is the stress concentration factor and *D* is the average grain size [24,25]. In Equation (2), *b* is the Burgers vector in magnesium (=0.32 nm), *G_m_* is the shear modulus (=16.6 GPa), *λ* is the interparticle spacing, *d_p_* is the particle diameter (80 nm) and *V_p_* is the volume fraction of nanoparticles in the nanocomposite. In [11], the Orowan contribution of 1 wt.% AlN nanoparticles in an AM60 magnesium alloy was calculated to be 11.9 MPa. Other mechanisms, such as the load-bearing mechanism and the generation of geometrically necessary dislocations (GNDs) due to differences in the coefficient of thermal expansion (CTE) or the elastic modulus (EM) are ignored here, since calculations and estimations in [11] have shown that their contribution is negligible.

(1) $$\sigma_{y}=\sigma_{0}+k_{y}D^{-1/2}$$

(2) $$\Delta \sigma_{OR}=\frac{0.13bG_{m}}{\lambda }\ln\frac{d_{p}}{2b}$$

(3) where, λ=dp [(12Vp)13−1]

**Figure 7:**
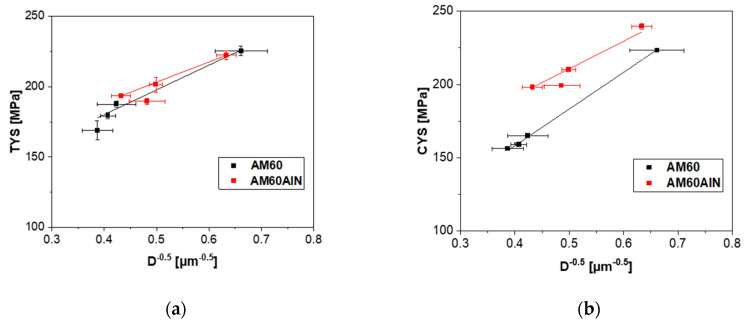
Hall–Petch plot of (**a**) tensile and (**b**) compressive yield strength over the reciprocal of the square root of grain size *D* following Equation (1).

Two Hall–Petch diagrams are shown in Figure 7, which present the relationship between the tensile (a) and compressive (b) yield strength (TYS and CYS) and the reciprocal of the square root of grain size *D* for AM60 and AM60AlN nanocomposites. Table 2 summarizes the $\sigma_{0}$ and $k_{y}$ of both AM60 and AM60 nanocomposites for tension and compression tests using the Hall–Petch relation.

A potential difference in the slope $k_{y}$ of both materials in tension as well as in compression is not clearly distinguished due to the large standard deviations (based on the limited number of samples). Thus, a change in the addressed grain-boundary-related strengthening is not revealed due to particle addition while not excluded. Still, the onset of the linear fits $\sigma_{0}$ in TYS seems to be slightly higher for AM60AlN, which is well within an expected gap of the envisioned 11.9 MPa [11]. In contrast, along with the compression tests and the same considerations along CYS, the onset $\sigma_{0}$ is visibly higher for the reinforced material and distinctly exceeds the envisioned 11.9 MPa as a result of particle strengthening, i.e., based on an Orowan mechanism of dislocation–particle interaction. In the considerations of grain boundary strengthening as applied by the Hall–Petch relationship, randomly textured samples were also not likely to reveal such differences between tensile and compression testing.

Considerations of the nature of the difference apply to explain the high impact that the nanoparticles have on CYS but not on TYS. A different activation ability of deformation mechanisms is based on the texture of the samples. Furthermore, the texture varies with the increase in the extrusion speed as a result of the developing microstructure. Even if the textures of the materials in this study partly remain weak, they develop with a certain tendency to align basal planes of the HCP lattice parallel to the extrusion direction; see Figure 4, where the significance of this orientation varies with the extrusion speed. It has been shown that such a texture suggests extension twinning as an important deformation mechanism when compressive stress is applied along the extrusion direction but not if tensile stress is applied [26,27,28]. An unrecrystallized fraction of the microstructure develops a stronger alignment. Thus, for such textured materials, tensile testing is mainly slip dominated, while during compression testing twinning is preferred. As twinning also leads to a specific strain hardening behavior with S-shaped stress–strain curves [27], this behavior is evident when comparing the results in Figure 5. As the determining mechanisms for yielding, it is likely that the impact of the AlN particles on the twinning mechanism causes the distinct increase in CYS shown in Figure 7b. It needs to be effective at the onset of yielding, thus at the nucleation phase of the twin development. 

## 5. Conclusions

The production of an AM60 Mg alloy with 1 wt.% AlN reinforcements using high shear dispersion technique and the further processing by extrusion with various ram speeds were successful. 

As the ram speed increases, the AM60-based samples obtain a fully recrystallized microstructure during extrusion, while the nanocomposites demonstrate a total recrystallized microstructure at low extrusion rates. The difference in the microstructure of both series is well depicted in the texture development as well, where a strongly pronounced component is found around the prismatic pole indicating a partially recrystallized microstructure that is visible in the AM60 alloy samples. This prismatic component decreases in intensity as the extrusion ram speed increases until it reaches the maximum speed at 8.0 mm/s. Further, in the AM60-AlN nanocomposites, this component is no longer visible, and there is no other pronounced component but only a further gradation of the texture. 

The AlN nanoparticles refined the AM60 alloy in almost all extrusion conditions, except the slowly extruded sample at 0.6 mm/s, which reveals similar grain size but a very different microstructure. The extensive recrystallized behavior, which is found in the AM60AlN sample extruded at 0.6 mm/s, is the result of an increase in the grain nucleation rate. 

According to the mechanical properties, the samples with the slowest extrusion speed at 0.6 mm/s revealed the highest stress values in tension and compression tests. Likewise, the addition of the nanoparticles reinforced the nanocomposites in almost all extrusion conditions. A distinct impact on the fracture strain was developed in tension after the entry of the nanoparticles as the AM60AlN-based series no longer exhibited ductile behavior. The contributions of Hall–Petch and Orowan indicated that the AlN nanoparticles strengthen the AM60 alloy. The yield strength values in tensile testing attributed to dislocation movement are slightly increased for the nanocomposites compared to the AM60 alloy. This is consistent with the assumption that the Orowan strengthening is effective and is around 11.9 MPa. The yield strength values from compression test are influenced by the twin deformation. It seems that the dominance of twin formation is visibly reduced with the addition of the AlN particles at the beginning of plastic deformation.

## Figures and Tables

**Table 1:** Results of tensile and compression tests at room temperature. UTS: ultimate tensile strength; TYS: tensile yield strength; UCS: ultimate compressive strength; CYS: compressive yield strength.

Material	TYS (MPa)	UTS (MPa)	Fracture Strain (%)	CYS (MPa)	UCS (MPa)	Fracture Strain (%)
AM60_0.6	225 ± 3	310 ± 2	20.3 ± 1.8	223 ± 0.4	428 ± 5	9.2 ± 0.2
AM60_4.4	187 ± 2	291 ± 1	21.9 ± 1.1	165 ± 0.1	398 ± 4	10.0 ± 0.5
AM60_6.6	179 ± 2	285 ± 1	23.2 ± 1.5	159 ± 0.1	391 ± 2	10.4 ± 0.2
AM60_8.0	169 ± 7	285 ± 1	23.5 ± 1.0	156 ± 0.4	394 ± 3	10.5 ± 0.4
AM60AlN_0.6	223 ± 3	310 ± 2	10.4 ± 1.2	240 ± 2	412 ± 6	8.6 ± 0.2
AM60AlN_4.4	202 ± 5	303 ± 5	12.1 ± 2.3	210 ± 0.3	379 ± 4	8.3 ± 0.2
AM60AlN_6.6	190 ± 2	299 ± 2	13.9 ± 2.2	199 ± 0.3	388 ± 7	8.6 ± 0.3
AM60AlN_8.0	194 ± 1	297 ± 2	10.5 ± 0.8	198 ± 0.3	390 ± 8	8.9 ± 0.3

**Table 2:** Results of Hall–Petch relation (Equation (1)) for tension and compression tests of AM60 and AM60AlN alloys, where $\sigma_{y}$ is the yield stress, $\sigma_{0}$ is the friction stress that allows dislocations to move on slip planes in a single crystal in the absence of any strengthening mechanisms, $k_{y}$ is the stress concertation factor and *D* is the average grain size.

	TYS AM60	TYS AM60AlN	CYS AM60	CYS AM60AlN
$\sigma_{0}$ (MPa)	110 ± 11	131 ± 26	58 ± 5	117 ± 7
$k_{y}$ (MPa µm−1/2)	176 ± 23	139 ± 54	251 ± 13	188 ± 15

## Data Availability

Not applicable. Data are needed for further studies.
